# Engineering the battle: Design-specific analysis of stag beetle mandibles for combat efficiency

**DOI:** 10.1093/pnasnexus/pgaf205

**Published:** 2025-06-20

**Authors:** Nasif Bin Saif, Ramin J A Guilani, Shayan Ramezanpour, Arman Toofani, Sepehr H Eraghi, Geoff Goss, Chung-Ping Lin, Stanislav Gorb, Hamed Rajabi

**Affiliations:** Mechanical Intelligence (MI) Research Group, Bioscience and Bioengineering Research Centre, London South Bank University (LSBU), London SE1 0AA, United Kingdom; Mechanical Intelligence (MI) Research Group, Bioscience and Bioengineering Research Centre, London South Bank University (LSBU), London SE1 0AA, United Kingdom; Centre for Industrial Mechanics, Department of Mechanical and Electrical Engineering, University of Southern Denmark (SDU ), Sønderborg 6400, Denmark; Mechanical Intelligence (MI) Research Group, Bioscience and Bioengineering Research Centre, London South Bank University (LSBU), London SE1 0AA, United Kingdom; Mechanical Intelligence (MI) Research Group, Bioscience and Bioengineering Research Centre, London South Bank University (LSBU), London SE1 0AA, United Kingdom; Mechanical Intelligence (MI) Research Group, Bioscience and Bioengineering Research Centre, London South Bank University (LSBU), London SE1 0AA, United Kingdom; School of Engineering and Design, College of Technology and Environment, London South Bank University, London SE1 0AA, United Kingdom; Department of Life Science, National Taiwan Normal University, Taipei 11677, Taiwan; Functional Morphology and Biomechanics, Institute of Zoology, Kiel University, Kiel 24118, Germany; Mechanical Intelligence (MI) Research Group, Bioscience and Bioengineering Research Centre, London South Bank University (LSBU), London SE1 0AA, United Kingdom; School of Engineering and Design, College of Technology and Environment, London South Bank University, London SE1 0AA, United Kingdom

**Keywords:** biomechanics, combat behavior, evolutionary adaptation, finite-element analysis, mechanical performance

## Abstract

Stag beetle mandibles exhibit remarkable interspecific variation, reflecting adaptations for species-specific combat strategies. This study investigates the functional basis of this diversity by analyzing the mandibles of five species using high-resolution finite-element analysis and geometric morphometrics. We simulated four distinct combat maneuvers—squeezing, holding, lifting, and twisting—and quantified mechanical performance via stress and strain energy, under standardized conditions. Principal component analysis of mandible morphology revealed two major axes of variation linked to shape and tip complexity. Our results show that certain mandible designs exhibit superior mechanical efficiency in maneuvers typical of their known fighting behavior, particularly in squeezing and twisting, suggesting functional specialization. However, mismatches also emerged, indicating latent mechanical capabilities or limitations in behavioral data. An exploratory correlation analysis highlights links between tip complexity (principal component 2) and biomechanical performance. Lifting performance was almost consistent across species, suggesting that whole-body kinematics may dominate this action. This work offers new insights into the evolution of animal weaponry by demonstrating that mandible shape in stag beetles is often aligned with species-typical combat strategies but not strictly constrained by observed behavior. It further provides a quantitative framework for linking morphology and mechanical performance in biomechanically complex systems.

Significance Statement Animal weapons often evolve to match species-specific combat strategies, yet the relationship between form, function, and behavior remains poorly quantified. Here, we integrate finite-element analysis and geometric morphometrics to investigate how mandible shape affects mechanical performance across four combat maneuvers in stag beetles. We find that mandibles often perform most efficiently in maneuvers aligned with species-typical behavior—particularly squeezing and twisting—while lifting appears largely independent of shape. A correlation analysis reveals links between tip complexity and biomechanical efficiency. These findings demonstrate that morphological variation can encode functional specialization, while also suggesting hidden mechanical capacities not evident from observed behavior. Our study offers a quantitative framework for understanding how animal weaponry evolves under behavioral, mechanical, and developmental constraints.

## Introduction

Since Darwin, animal combat weapons have been a central topic in evolutionary biology (1). From the shield and horn of the long-extinct *Triceratops* to the giant tusks of African elephants, weapons have played an integral role in the natural world (2). The most widely accepted explanation for the diversity of such weapons is their role in combat, particularly in male–male competition (1). However, animal weapons are often multifunctional. Beyond combat, they may also serve in intimidation (e.g. elephant tusks) (3), sexual signaling (e.g. antlers in elk) (4), or feeding (e.g. talons in birds of prey) (5).

Both environmental and behavioral factors play critical roles in shaping weapon morphology. For example, in burrowing dung beetles, narrow tunnels constrain movement, favoring hornless males that rely on sneaking behaviors, while horned males dominate direct tunnel defense (6, 7). Beyond environmental constraints, weapon structure also correlates closely with combat strategy, as seen in ungulates (8). Adult males with short, stubby horns tend to adopt a stabbing strategy; those with robust, curved horns typically engage in ramming; and individuals with long, far-reaching horns often engage in wrestling or fencing behaviors.

Similarly, in insects, a wide variety of combat adaptations can be observed. In many species, weapons take on diverse shapes, sizes, and forms. Some, like praying mantises (*Mantodea*), use their scythe-like forelegs to ambush unsuspecting prey, whereas bombardier beetles (*Carabidae*) eject chemicals that trigger an exothermic reaction reaching ∼100 °C (9). Rhinoceros beetles (*Dynastinae*) use a long horn extending from the dorsal region of the head or prothorax to engage in combat (10). In contrast, stag beetles (*Lucanidae*)—a distantly related group within Scarabaeoidea—employ a wide variety of mandible morphologies as weapons (11–14). While many insects use their mandibles for diverse functions such as burrowing, cutting vegetation, or feeding, the mandibles of male stag beetles appear to be specialized almost exclusively for combat (15–18). Although stag beetles have been the subject of considerable research, the variation in mandible morphology across species, and how they are used in combat, has received relatively little attention (19–21).

**Fig. 1.**
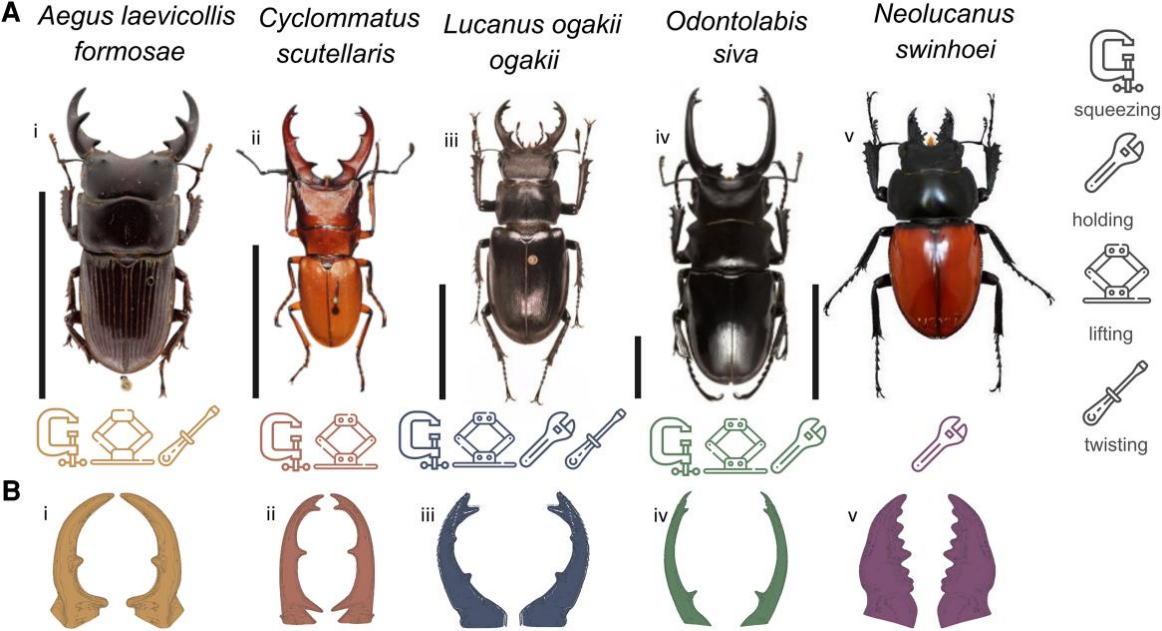
Studied stag beetles, mandibles, and their use in fights. A) Morphology of stag beetles’ species. i) *A. laevicollis* males use their mandibles to squeeze rivals, followed by lifting and twisting them. ii) *C. scutellaris* squeeze and immobilize rivals before lifting and toppling them, followed by tossing them to the ground via lift, leveraging their long mandibles. iii), iv) *L. ogakii* and *O. siva* hold and squeeze opponents, providing an effective grip for lifting and ultimately throwing rivals to secure victory. *L. ogakii* additionally use mandibles to twist rival's body. v) *N. swinhoei* features mandibles identical to their female counterparts. These mandibles lift have not been observed in combat; thus, we assume that they are not used as weapons to squeeze or twist rival males, but are adapted for holding food sources while feeding. Scale bars: 2 cm. B) 3D models of the mandibles of corresponding species.

Stag beetles represent an ideal system for studying weapon diversity. First, they exhibit remarkable interspecific variation in mandible morphology (Fig. 1A) (12, 22, 23). Second, males engage in combat on a variety of natural substrates—such as tree trunks, branches, soil surfaces, and cavities—and use their mandibles in different ways, which may select for qualitatively diverse fighting structures (12, 24, 25). Third, mandibles are used as weapons during combat with rival males for access to females and resources. However, there is no evidence that females choose males based on the shape or size of their mandibles (18, 25, 26); thus, female choice is unlikely to have an effect on the evolution of mandible form and function.

Rivalry between male stag beetles typically escalates through a series of activities that incorporate jousting with mandibles at the longest range possible, pushing and shaking with the mandibles, and finally interlocking mandibles so that the tips or teeth of the mandibles can apply force on the adversary. The victor in these battles typically tosses the loser from the fighting site—such as logs or tree branches—and secures access to exclusive mating opportunities with nearby females or to lucrative sap-feeding sites that attract females (15, 16, 27). These mandibles are reinforced by a thick sclerotized cuticle, which helps to mitigate serious structural damage during combat (28). This reinforcement has been linked to the prevalence of only superficial damage observed under scanning electron microscopy (16, 29, 30).

This study investigates the relationship between mandible form and function in five species of stag beetles by testing whether their designs are structurally adapted for specialized fighting styles. We use principal component analysis (PCA) to visualize key morphological variables and apply finite-element (FE) analysis to assess stress and strain energy in the mandibles. To evaluate functional specialization, we test mandibles from five behaviorally and morphologically diverse species in both species-typical and atypical combat maneuvers: holding, squeezing, twisting, and lifting. Our findings reveal how mandible shape and detailed structural features are adapted for distinct fighting strategies, providing deeper insight into evolutionary optimization in weapon morphology and structure–function coevolution.

## Materials and methods

### Specimens and measurements

A total of 40 specimens from five species were measured with eight individuals per species. These are among only a few stag beetle species for which reliable fighting behavior data are available in the literature (12, 20, 21, 31–34). Only adult individuals of the same morph were included to reduce potential variability caused by changes in size and shape.

Dried samples of the male stag beetles, *Aegus laevicollis formosae*, were obtained from the preserved samples of the Functional Morphology and Biomechanics Research Group, Kiel University. The specimens of *Cyclommatus scutellaris*, *Odontolabis siva*, *Lucanus ogakii*, and *Neolucanus swinhoei* were received from the Systematics and Evolutionary Biology Lab, Department of Life Science, National Taiwan Normal University. *N. swinhoei* is used as a control, as males and females have similar mandible morphology (34), lacking the sexual dimorphism characteristic of most other stag beetles. This absence of sex-specific differentiation makes it well suited for examining the functional implications of mandible shape. The mandibles of the selected species are employed in a diverse range of combat maneuvers, including squeezing, holding, lifting, and twisting (Fig. 1).

We measured morphological attributes that might play a role in their various combat style and how they utilize their mandibles. Morphological attributes included mandible length, presence of teeth, and the shape of mandibles tip (singular/forked), as these are the most observable and quantifiable traits. Where required, the observations were done on mandibles dorsal view using a light microscope Leica S9D equipped with a flexicam C3 camera (Leica Microsystems GmbH, Wetzlar, Germany) and the measurements of mandible-to-body ratios and teeth counting were taken using digital pictures and the program ImageJ (35). Each beetle's dorsal side was photographed, focusing on the mandibles, and pinned down. The specimens were placed over a grid that was used to calibrate measurements taken on the photographs.

### 3D modeling

To develop 3D models of mandibles (Fig. 1B), we scanned the specimens using a micro-CT scanner. We used the air-dried specimens, which were fixed on a specimen holder using carving wax. Dehydration of the specimens did not change the mandible shape because mandibles are made of highly sclerotized cuticle (36). The images were taken utilizing a Skyscan 1172 high-resolution µCT system Bruker micro-CT (Bruker Corporation, Kontich, Belgium) at an acceleration voltage of 45 kV, at a current of 250 µA, with a voxel size of 0.4 µm, and at an exposure time of 2 sec per projection. The scans were conducted at 360°. The raw data were processed and reconstructed using NRecon Reconstruction Software v.1.6.6 (Bruker), rendered using Fiji-ImageJ v.1.53q (37), and processed with Meshlab v.2022.02 (38). Both software packages are open access.

The mandible of each examined specimen was isolated from the rest of the specimen using the segmentation tool in Meshlab. Each mandible model was checked for holes, spikes, self-intersections, and nonmanifold edges, all of which are common errors while building 3D models from an image stack. The models were repaired using the Repair Module in Meshlab. The surface noise was reduced at a medium smoothness level using the Smooth Module.

### FE simulation of functional maneuvers

The FE method is a numerical method that can be used for the structural analysis of complicated structures such as insect mandibles. These structures are discretized into a large number of small elements (or mesh), for which stresses, strains, and displacements are calculated under defined loading conditions (39–41).

To evaluate the effect of shape on the biomechanical performance of the mandibles under different combat styles, FE analyses were performed using the software package ABAQUS (Simulia, Johnston, Rhode Island, USA). Only the mean standard model of each species was used to reduce complexity. We imported the solid models from Meshlab to ABAQUS. The mandible material was modeled to be linear elastic and isotropic, with a density of 1,200 kg/m^3^, an elastic modulus of 5.1 GPa, and a Poisson's ratio of 0.3. These material properties are the same as those measured for *Cyclommatus metallifer*. We used *C. metallifer* as standard due to it being the most studied stag beetle in terms of mechanical characterization (42).

It is important to note that the primary objective of this study was not to replicate the precise real-world behavior of mandibles, but rather to perform a comparative analysis to evaluate how differences in shape influence mandible functionality. This approach required controlling certain variables to isolate the effects of shape alone. To achieve this, all mandibles were scaled to have the same surface area and assigned identical material properties—those of *C. metallifer*. This methodological decision was essential to eliminate the influence of interspecific variations, allowing us to assess shape effects in isolation. Such simplifications are consistent with established practices in comparative biomechanics (40). Moreover, the use of *C. metallifer* properties is supported by experimental findings from Goyens et al. (42), which reported no significant difference in the elastic modulus between fresh and dried samples. This suggests that the mandibles are composed of highly sclerotized cuticle with relatively uniform mechanical properties (43–46).

We employed FE analysis to calculate the maximum principal stress and strain energy experienced by stag beetle mandibles during simulated combat maneuvers. Maximum principal stress is a widely accepted metric for predicting failure in brittle, nonmetallic materials (39), making it particularly suitable for modeling the fracture observed in stag beetle mandibles (47). To ensure the robustness of our results, we identified and removed stress singularities—artificially high stress values arising from the modeling process that do not reflect actual mechanical behavior. These artifacts, if not excluded, could lead to erroneous interpretations. Following Dumont et al. (40), we implemented a proximity-based outlier detection method (30, 47), whereby an element group was classified as an artifact and excluded if it comprised fewer than 0.0025% of the total number of elements and exhibited similarly elevated stress values—defined as falling within a 10-MPa range of one another.

The simulations involved the mandibles interacting with cylindrical objects (Video S1), each measuring 2.76 mm in diameter. This specific size was chosen for two main reasons. First, it closely matches the average diameter of the mandibles themselves, making the simulations more realistic. Second, the size is representative of the typical objects these beetles might encounter and grab using their mandibles in their natural environment, such as body parts of opponent beetles during combat. The cylindrical shape is particularly suitable for this purpose because it effectively simulates the round, elongated segments commonly found in beetle anatomy, such as mandibles and legs. The chosen diameter strikes a balance, being large enough to be relevant for a range of potential interactions, yet small enough to reflect the physical constraints and challenges of grabbing opponents during actual combat.

The loading and boundary conditions applied to the mandibles and the object were tailored to reflect the mechanics of each maneuver, as described below and visualized in Fig. S1.

#### Squeeze

The object was positioned between the two mandibles, and a unit force was applied at the mandible bases—corresponding to the sites of muscle attachment—to simulate closing and compression. The mandibles were permitted to rotate about their articulations with the head, while the object was unconstrained except along its longitudinal axis, preventing motion in that direction.

#### Hold

The object was placed between the mandibles and brought into contact with both. A unidirectional pulling force was applied to the object, simulating an attempt to extract it from the mandibles’ grip. The mandibles were constrained to rotate about their joints with the head, and the object was restricted to movement only along the direction of the pulling force.

#### Lift

The object was positioned horizontally in contact with the dorsal surfaces of the mandibles. A unit downward force was applied to the object to simulate resistance experienced during the lifting of an opponent. The mandibles were fully fixed in space, and the object was allowed to move only downward—normal to the dorsal surface of the mandibles.

#### Twist

The object was placed as in the *squeeze* simulation, gripped between the mandibles. A rotational force was applied around the insect's longitudinal body axis, simulating the torsional forces experienced during body twisting, which translates into a torque.

We also made sure that the mandible's movement in all simulations was symmetric, thus ensuring that the mandible does not cross the symmetry plane. For meshing both the mandible and the object, we used CPS4R elements—a four-node bilinear plane stress quadrilateral, reduced integration, hourglass control. The mesh size was optimized through mesh convergence analyses to balance accuracy and computational efficiency.

To facilitate meaningful comparisons, the results—including stress and strain energy values—were normalized using min–max normalization, according to the following equation:


(1) $$X_{\mathrm{norm}}=\frac{X-X_{\min}}{X_{\max}-X_{\min}},$$

where *X* is the original value, $X_{\min}$ is the minimum value in that dataset, $X_{\max}$ is the maximum value, and $X_{\mathrm{norm}}$ is the resulting normalized value, which ranges between 0 and 1.

### Morphometrics

In our study, we analyzed the shape variation of beetle mandibles using a combination of anatomical landmarks and semilandmarks, as per the methods outlined by Tatsuta et al. (48). Anatomical landmarks are specific points on an organism that are prominent and are consistent across specimens. Semilandmarks, on the contrary, are points that help in defining shape but are not strictly based on biological homology.

For each mandible, we placed 10 anatomical landmarks and 260 sliding semilandmarks to accurately capture its shape (Fig. S2). Among these semilandmarks, 20 were placed along the curves at the base of the mandible and 240 were distributed evenly across its surface. We used the open-source software package SlicerMorph (49) for this landmark placement.

The semilandmarks on the curves were equally spaced and positioned between the anatomical landmarks to avoid bias in distance measurements, following guidelines from the literature (50, 51). The surface semilandmarks were manually digitized on a template mandible in SlicerMorph, and this template was then used to project these landmarks onto all examined mandibles. To ensure homogeneity in terms of the position of the landmarks, these semilandmarks were adjusted, or “slid,” minimizing the bending energy, and ensuring geometric consistency. This sliding was performed using the ALPACA package which facilitates automatic alignment (52).

The selection of 10 anatomical landmarks and 260 semilandmarks for analyzing mandible shapes in beetles is a methodical balance between precision and practicality. The 10 anatomical landmarks were chosen for their biological significance, ensuring consistency across different specimens. Meanwhile, the 260 semilandmarks allowed for a detailed mapping of the mandible's complex surface, capturing subtle but critical shape variations. This specific number strikes a practical balance, ensuring efficient data collection and processing, while adhering to standard research practices for comparative morphological studies. This approach is designed to yield a thorough and accurate representation of mandible shapes, facilitating meaningful biological comparisons and analyses.

We then conducted a generalized Procrustes analysis (GPA) using the GPA package in SlicerMorph to compare the shapes of the mandibles. The shape variations were visualized using PCA, performed on the aligned coordinates. The PCA helped us identify critical axes (principal components [PCs]) and the morphological features most influential on these axes. This process, akin to Horn's parallel analysis (53), allowed us to discern critical axes based on eigenvalue distributions.

To measure shape divergence among the examined mandibles, we computed Euclidean distances using the centroid scores from the first two PCs. We calculated these distances using the formula:


(2) $$D\;=\;\sqrt{{\left(x_{2}-x_{1}\right)}^{2}+{\left(y_{2}-y_{1}\right)}^{2}},$$

where $x_{1}$ and $y_{1}$ are the centroid scores of the first mandible on PC1 and PC2, respectively, and $x_{2}$ and $y_{2}$ ​ are the centroid scores of the second mandible on PC1 and PC2.

**Fig. 2.**
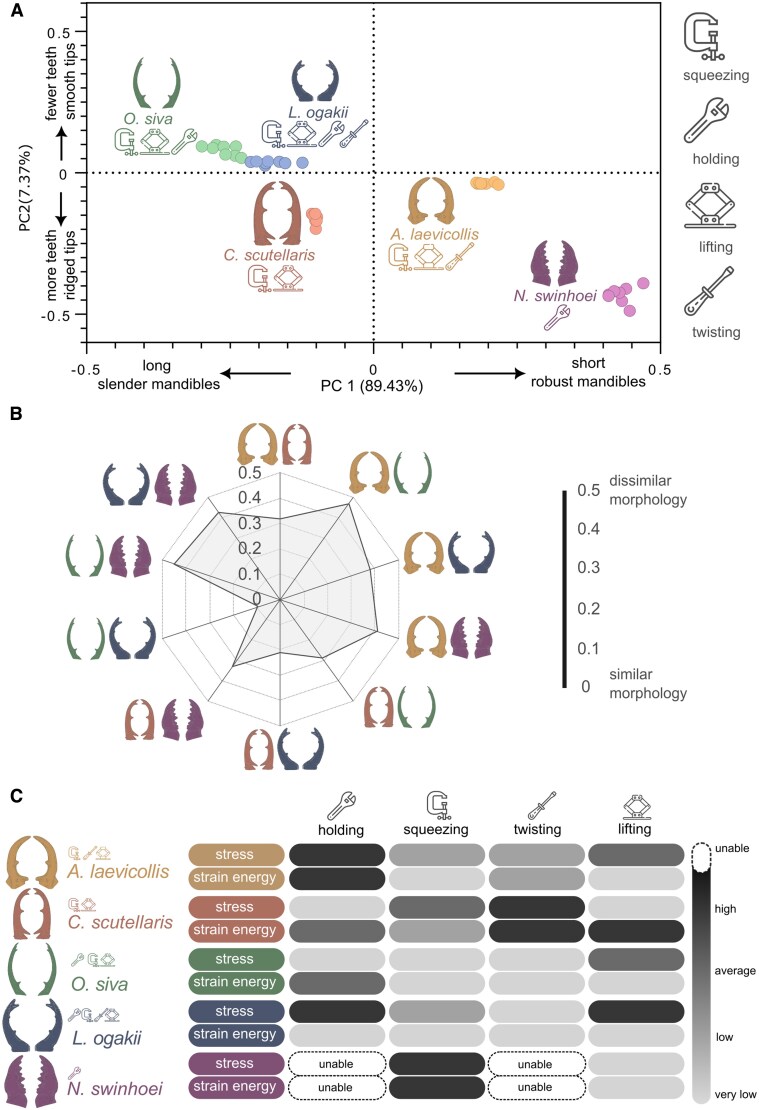
Biomechanical analysis of mandibles. A) PCA scatterplot of the five species studied, including *O. siva*, *L. ogakii, C. scutellaris, A. laevicollis*, and *N. swinhoei*, respectively. Data points for each species are grouped into distinct clusters. PC1 mainly reflects the major variation in mandible shape, while PC2 captures differences in fine structural features. B) Pairwise Euclidian distance between the five selected species. C) Comparison of the stress and strain energy between the models in different maneuvers. “unable” indicates that the mandible was unable to perform a maneuver in our simulations. The categories “very low,” “low,” “average,” and “high” correspond to normalized values in the ranges 0–0.25, 0.25–0.5, 0.5–0.75, and 0.75–1.

Lastly, we determined the geometric mean shape of the mandibles for each species, visualized on a scatter plot of PC1 vs. PC2 (Fig. 2A). We also conducted single-factor ANOVA test with PC1 and PC2 to assesses whether there are statistically significant differences in the mandible shapes among different species. The ANOVA results showed statistically significant differences in mandible morphology among beetle species for both PC1 (*P* < 0.02) and PC2 (*P* < 0.04).

### Correlation between morphology and biomechanics

To explore the potential relationship between mandible shape and biomechanical performance, we performed an exploratory Pearson correlation analysis. Specifically, we assessed the association between morphological variation—captured by PC1 and PC2 scores from the PCA—and mechanical outputs (i.e. maximum principal stress and strain energy) for each simulated maneuver. This allowed us to identify whether specific shape features were consistently linked to improved or reduced performance across maneuvers.

Given the small number of species examined (*n* = 5), this analysis was intended as a hypothesis-generating tool rather than a definitive test of correlation strength. Correlation coefficients (*r*) and corresponding *P*-values were calculated using standard formula below, and all results are presented in Table S1.


(3) $$r=\frac{\sum_{i=1}^{n}\;(x_{i}-\bar{x})(y_{i}-\bar{y})}{\sqrt{\sum_{i=1}^{n}\;{\left(x_{i}-\bar{x}\right)}^{2}\sum_{i=1}^{n}\;{\left(y_{i}-\bar{y}\right)}^{2}}}$$

where *n* is the number of pairs of data points, $x_{i}$ is the individual values of the first variable, $y_{i}$ is the individual values of the second variable, $\bar{x}$ is the mean of all the *x* values, and $\bar{y}$ is the mean of all the *y* values.

## Results

### Comparison of mandible morphology among species

We examined the mandibles of five beetle species: *A. laevicollis formosae, C. scutellaris, O. siva, L. ogakii,* and *N. swinhoei*. There are notable differences in mandible morphology across these species. For example, *A. laevicollis*'s mandibles are blunt, short, and conical with an end curve, distinctly differing from *C. scutellaris*'s elongated, forked mandibles. The quantification of shape variation was done utilizing PCA. The first two PCs accounted for a significant portion of the variation among the species, with 89 and 7.4%, respectively. These components were plotted (Fig. 2A), revealing the variations along two axes and including 90% equal frequency ellipses, which contained ∼93% of the data points for each group. This scatter plot suggests that the divergence observed in PC1 is not merely due to differences in aspect ratio but also in the shape of the teeth.

Using Procrustes distance to measure shape deviation, the study revealed varying degrees of shape divergence among species (Fig. 2B). The pairwise Euclidean differences of centroid scores on PCs indicated that *O. siva* and *L. ogakii* have the least shape divergence among them, while the greatest divergence is observed between *A. laevicollis* and *O. siva*, closely followed by *O. siva* vs. *N. swinhoei.* A single-factor ANOVA was conducted on the landmark data, yielding a *P*-value of 0.01, indicating significant differences in mandible morphology among the five species.

The observed differences are not uniform across species, suggesting potential evolutionary adaptations in mandible design for each species. PC1 captures the major variation in mandible shape, ranging from longer, slender mandibles with forked tips and fewer, larger teeth (more negative PC1 scores), to shorter, more robust mandibles with a lower length-to-base ratio, a single-tip structure, and a greater number of smaller marginal teeth (more positive PC1 scores). PC2 primarily reflects variation in the fine structural features of the mandible tip. Negative PC2 scores are associated with more teeth and pronounced ridges or denticles within the forked region, while positive PC2 scores correspond to a reduction in the number of teeth and smoother, less ornamented tip morphology.

### Comparison of mandible biomechanics among species

We measured the stress and strain energy experienced by the mandibles during various maneuvers (Video S1). The normalization involved scaling the stress and strain energy values relative to the highest value being 1 and the lowest value being 0, allowing for a direct comparison of biomechanical properties across different mandible designs and maneuvers (Figs. 2C and S3).

High stress values in a mandible during a specific maneuver indicate that certain regions experience disproportionately large forces. This localized concentration of stress may signal an increased risk of damage or failure under extreme loading. Conversely, lower stress values suggest a more even distribution of mechanical loads, which could imply that the mandible's geometry is well suited to that particular action, reducing the likelihood of material failure. In a comparative framework where both surface areas of mandibles and their material properties are standardized, lower peak stresses may reflect more robust or mechanically optimized designs for combat-related functions.

Strain energy quantifies the elastic energy stored within a structure during deformation and reflects the combined effects of internal stress and displacement. In our models, where material properties were standardized across species, variation in strain energy arises primarily from geometric differences. Mandibles exhibiting higher strain energy were interpreted as less mechanically efficient in the context of force transmission, as they deform more under load and absorb energy that would otherwise be delivered to the opponent. Such energy dissipation is suboptimal for structures functioning as combat tools, where rigidity and effective force transfer are advantageous.

The results of our FE simulations, presented in Figs. 2C and S3, revealed substantial variation in stress and strain energy across species and maneuvers. By comparing these values within and between mandibles, we assessed the biomechanical performance of each design under distinct functional demands. For interpretative clarity, we refer to stress and strain energy values using the following categories: *very low* (0–0.25), *low* (0.25–0.5), *average* (0.5–0.75), and *high* (0.75–1.0), based on normalized scales.

*A. laevicollis* performed best during squeezing, twisting, and lifting, exhibiting very low or low stress and strain energy in all three, except for moderately elevated stress during lifting. However, during holding—the only atypical maneuver for this species—it showed both high stress and high strain energy, indicating poor mechanical performance and a heightened risk of structural failure. *C. scutellaris* did not perform exceptionally well in any maneuver but showed generally average performance in holding, squeezing, and lifting. Its performance during twisting, an atypical maneuver, was notably poor, with both stress and strain energy reaching high levels. *O. siva* showed strong overall performance, particularly in squeezing (a typical maneuver) and twisting (an atypical one), both characterized by very low stress and strain energy, indicating high mechanical efficiency. *L. ogakii* was the only species to exhibit exceptionally low strain energy across all maneuvers—each considered typical for this species. However, it experienced high stress during holding and lifting. *N. swinhoei*, which lacks clearly defined typical maneuvers, failed to retain the object during holding and twisting simulations due to slippage under the imposed boundary conditions. It also performed poorly in squeezing, with both stress and strain energy reaching high levels. Interestingly, it performed surprisingly well in lifting, showing low stress and strain energy.

### Correlation between mandible morphology and biomechanical performance

An exploratory Pearson correlation analysis was conducted to assess relationships between mandible morphology (PC1 and PC2) and mechanical performance (stress and strain energy) across the different simulated maneuvers. Given the small sample size (*n* = 5), most correlations—particularly those involving PC1—did not reach statistical significance, though this may reflect either limited statistical power or a true lack of association. In contrast, some significant correlations were observed for PC2. Notably, higher PC2 scores were associated with lower stress during squeezing (*r* = −0.96, *P* < 0.05), as well as lower stress (*r* = −0.98, *P* < 0.05) and strain energy (*r* = −1, *P* < 0.01) during twisting. Full correlation results are presented in Table S1.

## Discussion

This study investigated the functional relationship between mandible shape and biomechanical performance in five species of stag beetles, each exhibiting distinct combat behaviors. By integrating morphometric and FE analyses, we tested whether interspecific differences in mandible morphology correspond to mechanical performance under species-typical and atypical combat maneuvers. Our results revealed that certain mandible designs exhibited lower stress and strain energy in maneuvers characteristic of their species-typical fighting styles. However, this pattern was not universal, highlighting the complexity of structure–function relationships in systems shaped by both mechanical and behavioral constraints.

Among the maneuvers tested, lifting emerged as a biomechanical outlier: performance in this action showed low stress and strain energy across nearly all species, including *N. swinhoei*, which is not known to engage in combat. This uniformity suggests that lifting may be less sensitive to specific mandible morphology and more dependent on coordinated body-level kinematics—such as head tilting and leg propulsion—as documented in behavioral studies (12). Alternatively, the broadly low biomechanical demands of lifting may have favored the evolution of convergent mandible features among species that regularly perform this maneuver. In contrast, twisting appears more mechanically demanding and shape-sensitive. Species such as *A. laevicollis* and *L. ogakii*, which are known to use twisting in combat, exhibit relatively low stress and strain energy during this action, whereas *C. scutellaris* performs poorly and *N. swinhoei* is unable to perform it altogether. *O. siva* demonstrates unexpectedly high mechanical performance in twisting—a maneuver for which no behavioral evidence currently exists—suggesting that biomechanical potential may exceed observed behavioral patterns. This mismatch likely stems from either undocumented behaviors or a broader mechanical design that supports versatility beyond observed usage. Together, the performance patterns suggest that mandible morphology may be broadly aligned with species-typical fighting behaviors—a pattern also observed in beetle horns, where structural performance tends to match typical loading regimes (27).

The observed variation in biomechanical performance across maneuvers aligns with key axes of morphological divergence identified through PCA. Our exploratory Pearson correlation analysis revealed that higher PC2 scores—corresponding to smoother, less ornamented tips—were significantly associated with reduced stress and strain energy in twisting and squeezing maneuvers. This suggests that tip complexity may influence mechanical performance in tasks requiring force transmission or rotational stability. While the small sample size limits statistical power, the strength and direction of these associations point to PC2 as a potentially important morphological axis governing functional specialization. Notably, species with low PC2 scores, such as *L. ogakii*, which possesses prominent denticles and a deeply forked tip, perform exceptionally well in twisting. Intriguingly, *O. siva*—which also performs well in twisting despite no behavioral evidence for this maneuver—shares a similar overall tip architecture. These results support the idea that shape variation captured by PC2 reflects functional specialization rather than superficial diversity—and may reveal biomechanical capacities not currently expressed behaviorally. Building on this, our broader findings point to a more complex relationship between morphology and combat behavior across species, suggesting that structural and behavioral evolution is not always tightly coupled.

While our findings suggest that mandible morphology often aligns with species-typical combat performance, several patterns point to a more complex relationship between structure and behavior. An additional dimension worth considering is the potential decoupling between behavioral expression and morphological divergence. For instance, species that exhibit similar fighting behaviors may differ substantially in mandible shape, while others with comparable morphologies may employ distinct combat strategies. This disconnect implies that behavioral evolution may proceed more rapidly or flexibly than morphological change—possibly due to the semiindependent developmental regulation of mandible traits by appendage-patterning genes (54), or due to functional tradeoffs imposed by the mandibles’ roles in feeding or substrate interaction. Supporting this, *Aegus chelifer chelifer* populations show morph diversity with different allometric intercepts between body and mandible size across habitats (urban vs. forest), suggesting that ecological context can also influence weapon design (55). Taken together, these findings reinforce the idea that structure–function relationships are modulated by both behavioral demands and environmental or developmental constraints. Comparative studies incorporating more species and detailed behavioral datasets—particularly in regions such as Taiwan, where lucanid subspeciation and morphological variation are prominent (56)—may help clarify the extent to which modular or decoupled evolution shapes beetle weaponry.

Our models applied symmetric boundary conditions and enforced symmetrical mandible movement across the sagittal plane to simplify the complex, often asymmetric loading scenarios encountered during real combat (57). This constraint prevented the mandibles from overlapping—a phenomenon observed in some natural behaviors, particularly during twisting and gripping. As a result, the movement constraints imposed in our simulations may have prevented certain kinematic interactions, which could alter force application or enhance gripping stability in real contests. Future studies incorporating asymmetric movement, nonuniform loading, and dynamic simulation will be crucial to capture more realistic interaction forces and evaluate how asymmetry contributes to combat efficiency.

Another important methodological consideration is our assumption of uniform material properties across species. While this approach allows for isolating shape-based performance differences and aligns with prior comparative biomechanics studies, it necessarily simplifies the real-world interplay between structure and substance. Differences in cuticle composition, material heterogeneity, or local reinforcement (e.g. sclerotization gradients) could significantly affect mandible mechanics and fracture resistance. Such gradients in mechanical properties have been frequently observed in insect mouthparts, including the mandibles of termites (58), trichopteran larvae (59), and praying mantises (60). However, to our knowledge, no studies have systematically investigated property gradients in stag beetle mandibles, and even baseline mechanical measurements remain scarce (42). Integrating species-typical and spatially resolved material characterization into future models will provide a more comprehensive understanding of how biological materials and geometry coevolve to support combat performance.

## Conclusion

This study reveals that mandible morphology in stag beetles is broadly tuned to support species-specific combat strategies, especially in high-demand maneuvers such as twisting and squeezing. At the same time, several mismatches between morphology and observed behavior suggest that functional capacity may extend beyond known behavioral patterns—whether due to latent biomechanical potential, behavioral plasticity, or observational gaps. The finding that lifting performance is largely independent of morphological variation further points to the integration of mandibles within a whole-body combat system. Moving forward, a deeper understanding of animal weaponry will require the integration of dynamic biomechanical modeling, real-time behavioral analysis, and species-specific material characterization. Such efforts will not only refine our understanding of evolutionary biomechanics but also inspire the design of more versatile, adaptive technologies in soft robotics and bioinspired engineering.

## Supplementary Material

pgaf205_Supplementary_Data

## Data Availability

The authors declare that the data supporting the findings of this study are available within the paper and its supplementary information files.
