# Supplementary material for: Engineering the battle: Design-specific analysis of stag beetle mandibles for combat efficiency
Source: PNAS Nexus. 2025 Jun 20;4(7):pgaf205. doi: 10.1093/pnasnexus/pgaf205 (PMC12231608; doi:10.1093/pnasnexus/pgaf205)
Supplement: pgaf205_Supplementary_Data [file pgaf205_supplementary_data.zip › PNASNEXUS-PNASNEXUS-2025-00111R-s01.pdf]

Engineering the Battle: Design-Specific Analysis of Stag Beetle Mandibles for Combat Efficiency

Nasif Bin Saif[1], Ramin J. A. Guilani[1,2], Shayan Ramezanpour[1], Arman Toofani[1], Sepehr H. Eraghi[1], Geoff Goss[3], Chung-Ping Lin[4], Stanislav Gorb[5], Hamed Rajabi[1,3]

[1] Mechanical Intelligence (MI) Research Group, Bioscience and Bioengineering Research Centre, London South Bank University, London, UK

[2] Mechatronics and Centre for Industrial Mechanics, Department of Mechanical and Electrical Engineering, University of Southern Denmark, SDU, Sønderborg, Denmark

[3] School of Engineering and Design, College of Technology and Environment, London South Bank University, London, UK

[4] Department of Life Science, National Taiwan Normal University, Taipei, Taiwan

[5] Functional Morphology and Biomechanics, Institute of Zoology, Kiel University, Kiel, Germany

correspondence

Correspondence to Hamed Rajabi at rajabijh@lsbu.ac.uk

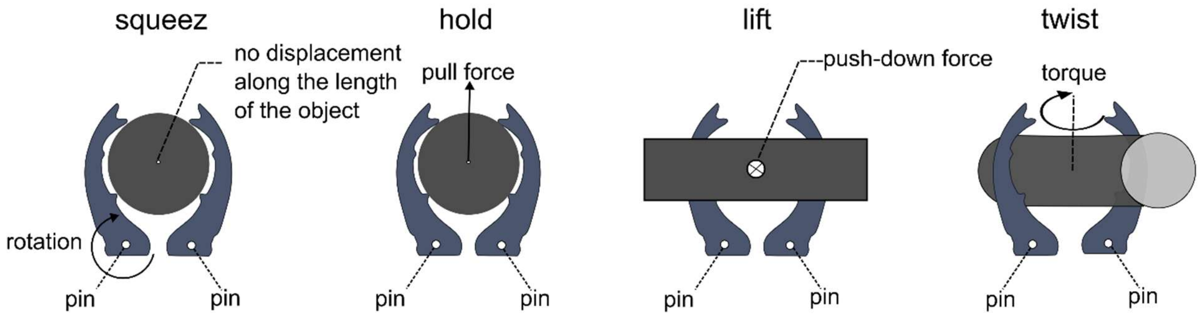

Fig. S1. Schematic drawing of the loadings and boundary conditions in the finite element analysis in four different manoeuvres. These are 2D drawings of the 3D simulations. The manoeuvres include *Squeeze*: The object is positioned between the mandibles, and a unit force is applied at the mandible bases to simulate closure. The mandibles are allowed to rotate about their joints with the head, while the object is constrained from moving along its longitudinal axis.
Hold: A pulling force is applied to an object held between the mandibles, simulating extraction resistance. Mandibles rotate about their joints, and the object moves only in the direction of the applied force.
Lift: The object rests horizontally on the dorsal surfaces of the mandibles, with a downward force applied to simulate the lifting of an opponent. Mandibles are fixed in place, and the object is constrained to move only downward, perpendicular to the mandibles' upper surface.
Twist: The object is clamped between the mandibles, and a rotational force is applied about the insect’s longitudinal body axis to simulate body twisting. This applies a torque.

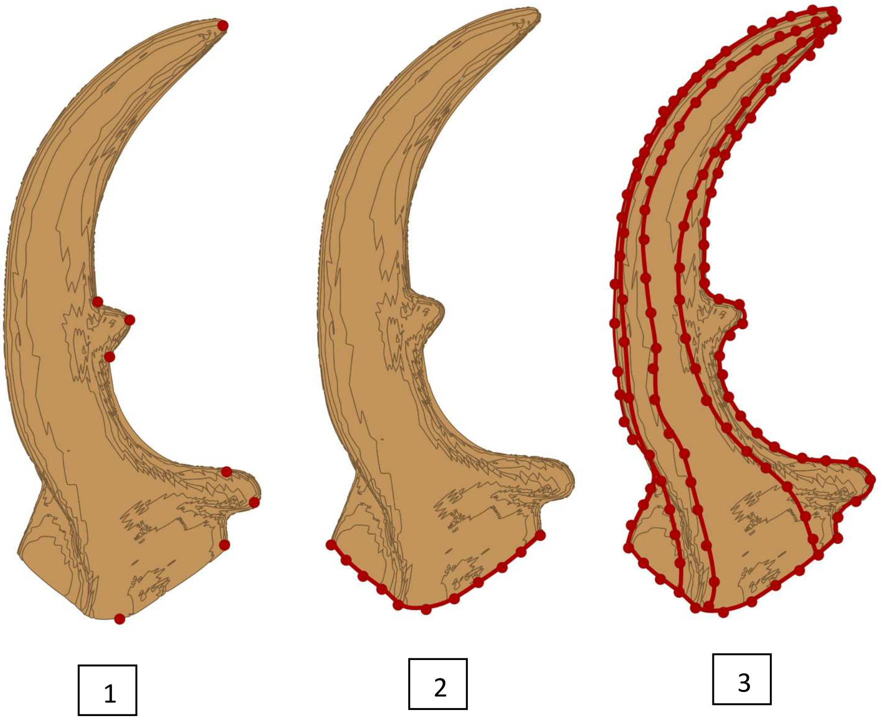

Fig. S2. Landmarks placed on mandible of *A. laevicollis.* It consists of 10 anatomical landmarks (1), 20 curved semi- landmarks bordering the base of the mandible (2), and 240 surface semi-landmarks distributed homogenously over the mandible surface (3).

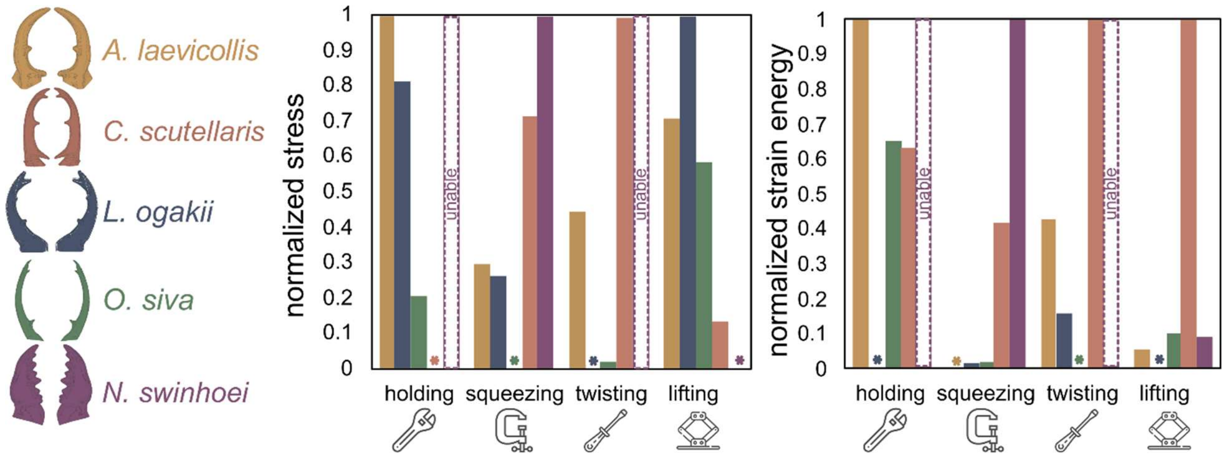

Fig. S3. Normalized stress and strain energy values of the mandibles in different manoeuvres. 0 indicates lowest value, 1 indicates highest value. Stress and strain energy are separated by manoeuvres. Empty bars indicate the mandible being unable to perform said function during simulation. The symbol * indicates value of zero. *N. swinhoei* failed to perform the 'holding' and 'twisting' manoeuvre in the simulation.

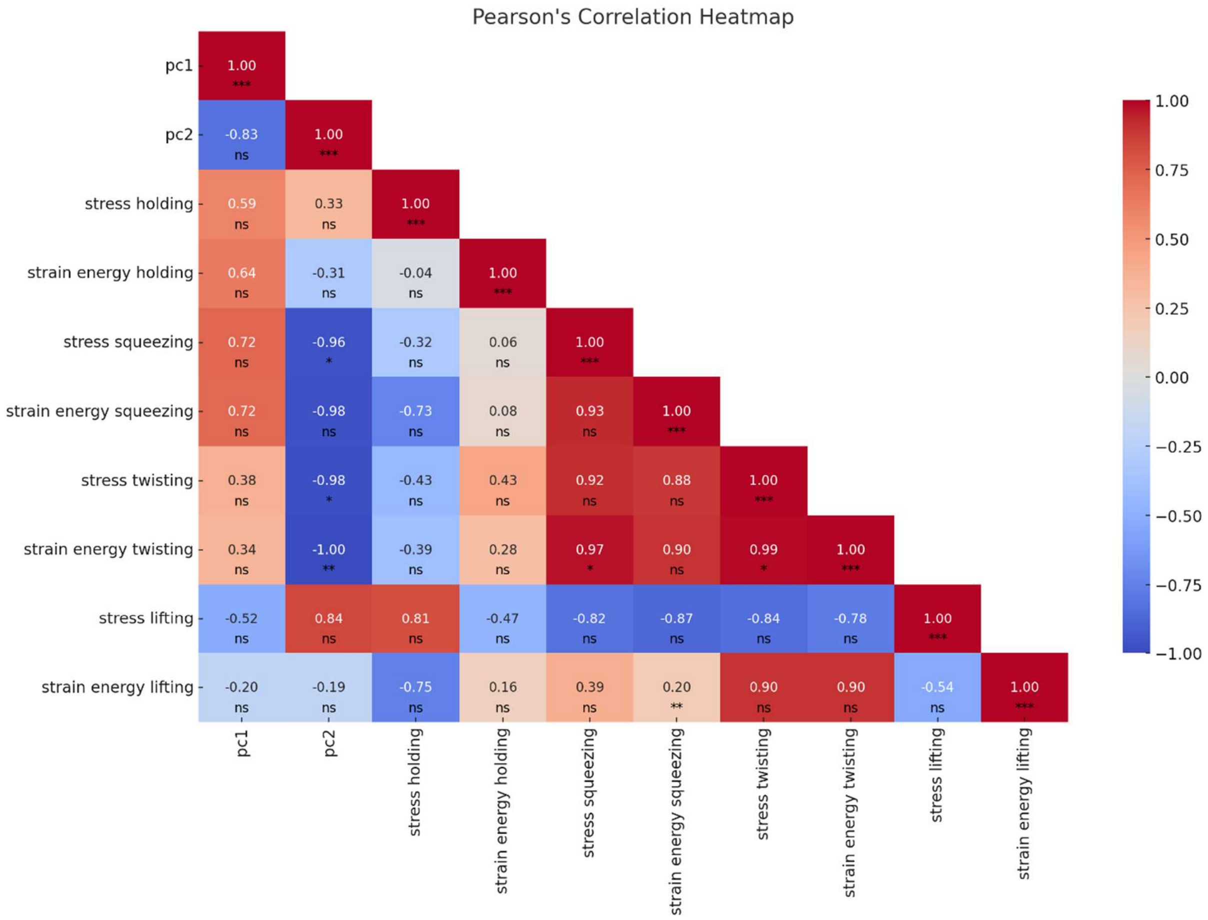

Table S1. Pearson's correlation heatmap. The results show the correlation between mandible morphology (characterized by PC1 and PC2) and biomechanical performance (characterized by stress and strain energy) in holding, squeezing, twisting and lifting. ns, $p >= 0.05$; *, $p < 0.05$; **, $p < 0.01$; ***, $p < 0.001$.

Video S1. Finite element simulation of the holding manoeuvre for *Lucanus ogakii*.
